# Chromosomal Microarrays in Prenatal Diagnosis: Time for a Change of Policy?

**DOI:** 10.3390/microarrays2040304

**Published:** 2013-12-05

**Authors:** Peter Miny, Friedel Wenzel, Sevgi Tercanli, Isabel Filges

**Affiliations:** 1 Medical Genetics, Department of Biomedicine, University Hospital Basel, Burgfelderstr. 101, Building J, CH-4055 Basel, Switzerland; E-Mails: friedel.wenzel@usb.ch (F.W.); isabel.filges@unibas.ch (I.F.); 2 Ultraschall Freie Strasse, Freie Strasse 38, CH-4001 Basel, Switzerland; E-Mail: sevgi.tercanli@unibas.ch; 3 Department of Medical Genetics, Box 153, BC Children’s and Women’s Hospital, 4480 Oak Street, Vancouver BC, V6H 3V4, Canada

**Keywords:** microarrays, array CGH, prenatal diagnosis

## Abstract

Microarrays have replaced conventional karyotyping as a first-tier test for unbalanced chromosome anomalies in postnatal cytogenetics mainly due to their unprecedented resolution facilitating the detection of submicroscopic copy number changes at a rate of 10–20% depending on indication for testing. A number of studies have addressed the performance of microarrays for chromosome analyses in high risk pregnancies due to abnormal ultrasound findings and reported an excess detection rate between 5% and 10%. In low risk pregnancies, clear pathogenic copy number changes at the submicroscopic level were encountered in 1% or less. Variants of unclear clinical significance, unsolicited findings, and copy number changes with variable phenotypic consequences are the main issues of concern in the prenatal setting posing difficult management questions. The benefit of microarray testing may be limited in pregnancies with only moderately increased risks (advanced maternal age, positive first trimester test). It is suggested to not change the current policy of microarray application in prenatal diagnosis until more data on the clinical significance of copy number changes are available.

## 1. Introduction

Microarrays detecting copy number changes in genomic DNA [1] have replaced conventional microscopic chromosome analyses in the routine diagnostic work-up of children and adults with suspected unbalanced chromosome anomalies during recent years [2,3]. The conventional approach is now restricted to the confirmation of clinically distinct chromosomal syndromes (e.g., Down syndrome) and the diagnosis of balanced structural anomalies in potential carriers such as family members at risk and couples with recurrent abortions or infertility. The reasons for this rather rapid paradigm change include a greatly increased resolution of this whole genome approach which allowed the definition of a number of previously unknown microdeletion or -duplication syndromes [4,5,6], but also the prospect of an easier standardization and finally automation of lab procedures as well as diagnostic assessment.

Although equal benefits may be expected for the prenatal diagnosis of chromosome anomalies the conventional microscopic approach has maintained its role as the “gold standard” for the time being [7,8]. The use of microarrays in this setting has been met with caution for various reasons [9,10]. These include the common observation of copy number variation (CNV) with unknown clinical significance (VOUS) being obviously more difficult to manage in the sensitive prenatal setting as well as the fact that the majority of all unbalanced chromosome findings in prenatal diagnosis concern common trisomies or other anomalies perfectly amenable to the conventional diagnostic approach or by the recently introduced non-invasive prenatal testing (NIPT) on a maternal blood sample [11]. This latter method uses high throughput sequencing technology on cell free DNA (cf DNA) fragments in the maternal plasma to assess the copy number of the most prevalent fetal aneuploidies currently including chromosomes 13, 18, 21, X and Y. Considerable attention, including extensive discussions in the lay press, has been paid to NIPT since the longstanding “dream” of a prenatal genetic diagnosis without physical risks for fetus and mother finally came true. NIPT currently dominates public discussions on prenatal diagnosis.

This contribution will review the current clinical application of microarrays in prenatal cytogenetics and discuss aspects relevant to its formal implementation as an addition to or as a replacement of alternative diagnostic options.

## 2. Procedures and Methods

### 2.1. The Current Approach to Prenatal Diagnosis of Chromosome Anomalies: A Brief Introduction

For decades advanced maternal age (in practice mostly >34 years) has been the predominant indication for amniocentesis (AC) or chorionic villus sampling (CVS) in order to obtain cells required for a cytogenetic work-up. A procedure-related risk for pregnancy loss of 0.5–1% is usually quoted for both these invasive procedures, but is likely to be significantly lower in experienced centers today [12]. Over the years the microscopic chromosome analysis has been amended with various molecular extensions for rapid aneuploidy testing or the targeted diagnosis of selected submicroscopic structural changes (fluorescence *in situ* hybridization (FISH) [13]; QF-PCR [14], MLPA [15]). For around 20 years, the additional assessment of maternal serum parameters and the more recent sonographic measurement of the thickness of the fetal nuchal skin (nuchal translucency) has greatly improved the pre-procedural risk assessment. The current first trimester test according to the Fetal Medicine Foundation London [16] or Germany [17] is the most popular among various proposed risk assessment schemes for all pregnancies regardless of maternal age. The sensitivity for trisomy 21 is around 90% for a false-positive rate of 5%. The implementation of this improved so-called risk “screening” has led to a significant decrease of invasive procedures [18]. 

The recently introduced NIPT, which is rather considered an advanced screening tool than a diagnostic test, offers a sensitivity above 99% for a false-positive rate of below 1% for trisomy 21 [19]. Referring to the still limited experience current recommendations suggest a conservative testing approach restricted to pregnancies at increased risk for common aneuploidies due to maternal age or first trimester test result [20,21,22]. Most experts agree, however, that NIPT will sooner or later replace the current risk assessment schemes except for the sonographic evaluation of the fetus and induce another dramatic decrease of invasive procedures.

Invasive testing remains the primary option of choice in pregnancies at high risk for chromosome abnormalities [12]. These are mainly identified by a first trimester test result in the high positive range or by fetal malformations and other anomalies assessed by ultrasound. A total of some 5–10% of all pregnancies will probably fall into this category. Specific congenital anomalies (such as, e.g., an omphalocele or heart defects), particularly in multiple occurrence or combined with intrauterine growth restriction, may pose a high risk for unbalanced chromosome counts including unusual structural changes and microdeletion or -duplication syndromes. In parallel to experiences in postnatal diagnostics, microarrays would be the subsequent diagnostic option in pregnancies with suggestive ultrasound findings but a normal conventional karyotyping result. A subgroup of couples, however, ready to accept the small procedure related risk, will seek a comprehensive exclusion of fetal chromosomal conditions irrespective of prior risk assessments. 

### 2.2. Technical Aspects

Following the seminal paper by Pinkel *et al.* [23] the diagnostic application of array CGH started with BAC clones and insert sizes in the 100–150 kb range targeted to genome regions of interest in cancer samples. The number of targets steadily increased and tiling path BAC arrays with 32,000 targets covering the whole genome began to be used in constitutional cytogenetics to search for cryptic chromosome anomalies [24]. In a further evolutionary step the large BAC inserts were replaced by synthetic oligonucleotides of sizes in the 50 base range resulting in a major leap in resolution with current target numbers of several millions.

In parallel single nucleotide polymorphism (SNP) arrays became available originally designed for genotyping patients in genome wide association studies for complex diseases. These platforms also detect copy number changes and due to the abundance of SNPs throughout the genome are able to provide high resolution coverage. Some current dedicated array designs for use in high resolution cytogenetics rely on a combination of SNPs and oligonucleotides in order to maintain the advantages of both approaches. For a more in-depth review of the technical aspects involved in the clinical use of microarrays see references [25,26]. 

### 2.3. Limitations of Clinical Relevance

Microarrays detect changes in copy number but not balanced chromosome anomalies such as translocations, inversions and others. While the copy number change provides the critical information relevant to the immediate management of the pregnancy it does not reveal its etiology which in a proportion of cases will be an unbalanced segregational product of a parental balanced rearrangement. This requires adequate follow-up studies using methods visualizing the chromosome structure such as conventional chromosome preparation and FISH. Ploidy changes such as triploidy or tetraploidy have been diagnosed with SNP arrays [27] but are not detectable by array CGH, a problem that can at least be partly solved by the use of abnormal control DNA (47, XXY) [28]. In principle, mosaicism will show with both approaches but the minimum level of detection is not clearly established and depends on technical as well as biological aspects [29], which is also true for conventional cytogenetics. 

The inadvertent detection of VOUS is the currently most significant objection against replacing conventional karyotyping with microarrays in all pregnancies undergoing invasive testing. There is an ongoing debate on the role of array design in terms of probe distribution (targeted *versus* genome wide) and the resolution attempting to optimize the trade-off between detection rate for clinically significant findings and the frequency of VOUS [9]. A current estimate of the mutation rates for large CNVs (>100 kbp) is 1.2 × 10^−2^ with a still unknown pathogenic proportion in fetuses [30]. Also mutation rates for smaller CNVs and their clinical impact have to be assessed in appropriate trials. Unsolicited findings with major clinical impact for the fetus and/or other family members such as predispositions for late onset disease as well as the management of CNVs with variable clinical expressivity and consequences are further issues of concern.

## 3. Results and Discussion

Numerous studies differ significantly in case numbers, patient ascertainment, design, and methodology and have addressed the central questions of interest: How often do microarrays show clinically significant copy number changes not detectable by conventional cytogenetics and how frequent are variants of unknown significance (VOUS). In a large collaborative trial of 29 US centers on more than 4,000 pregnancies at increased risk for chromosomal abnormalities due to advanced maternal age, first trimester test result, or abnormal ultrasound findings [31] microarrays detected all anomalies diagnosed by conventional cytogenetics except for balanced structural changes and triploidies as expected. In the subgroup of pregnancies with abnormal ultrasound findings and a normal conventional karyotype microarrays showed microdeletion or -duplications in 6%, half of these considered to be known pathogenic and the remaining potentially clinically significant, including those with variable clinical expressivity. The respective findings in the two lower risk groups were 1.7% with approximately 2/3 of these being potentially clinically significant. The overall rate of common benign copy number variation was around 30%.

From a single lab with more than 5,000 prenatal cases tested for various indications using platforms evolving with the technical progress a detection rate of 6.5% was reported for pregnancies with abnormal ultrasound findings and 8.2% after fetal demise [32]. VOUS were seen in 4.2% of all cases, a rate dropping to 0.4% if only *de novo* findings were included. 71% of the aberrations were not detectable by conventional karyotyping. The same group reported on risk stratification according to specific ultrasound findings in a retrospective study on almost 3,000 pregnancies [33]. Particularly high detection rates (10% or more) were observed in pregnancies with anomalies in two or more organ systems, and specific malformations including holoprosencephaly, posterior fossa defects, skeletal anomalies, ventricular septal defect, hypoplastic left heart, and cleft lip/palate occurring either isolated or combined with other anomalies. In a Spanish series of 276 pregnancies with fetal heart defects the overall rate of microscopically visible chromosomal pathology was high (15.9%) [34]. In a subset of pregnancies with a normal karyotype a targeted fluorescence *in situ* hybridization (FISH) test for 22q11 deletions resulted in an anomaly rate of 6.4%. Microarray testing in 51 patients with a normal karyotype and normal or no FISH result revealed one pathogenic copy number variant (2%) and no VOUS. 

Hillman *et al.* [35] reported an excess detection rate (aberrations detected in addition to conventional chromosome count) of 4.1% in their prospective cohort study on 243 pregnancies with abnormal ultrasound findings using a relatively low resolution BAC-based platform. The authors provide a systematical review and meta-analysis of relevant case series (totaling > 18,000 pregnancies) including the ones addressed above [31,32]. The overall excess detection rate was 10% and 7% for series published in more recent years (2011–2012). VOUS were observed in 2.1% of cases in the abnormal ultrasound group and in 1.4% with other indications. Another recent review of 18 series using different inclusion criteria [36] found an excess anomaly rate of 5.6% in 2,220 pregnancies with ultrasound anomalies in one anatomical system and 9.1% in 1,139 pregnancies with multiple anomalies. Similar detection rates were reported from a single center on 410 pregnancies [37]. VOUS were seen in 1.6% of all 1,115 cases. In a recent assessment of the clinical utility of microarray technologies [38] the review focused on more than 12,000 pregnancies with a normal conventional karyotype from various published series including some of those mentioned earlier. The rate of pathogenic copy number changes (pCNC) was 6.5% in pregnancies ascertained with an abnormal ultrasound, 1% with advanced maternal age and 1.1% with other indications (parental anxiety, abnormal serum screening and others). Series with abnormal ultrasound as an exclusive indication were considered separately, and an overall pCNC rate of 7% was found. The authors refrained from reporting VOUS rates because they considered the assessment conditions to be heterogeneous in the individual series.

### 3.1. Clinical Utility of Microarrays

**Figure 1:**
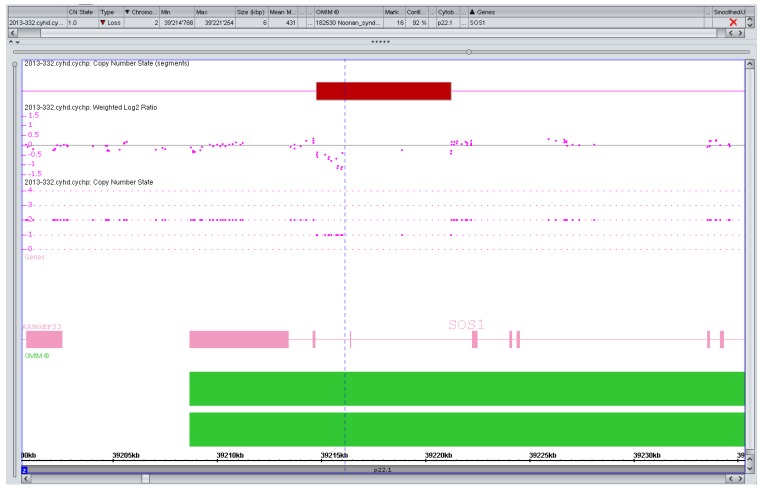
Artefact, variant of unknown significance (VOUS), or pathogenic copy number variation (CNV)? Markers left of the vertical line (n = 13) suggesting a small (1.2 kb) but intragenic (intronic) SOS1 deletion in a pregnancy with isolated increased nuchal translucency (>99. centile). SOS1 mutations are a known cause of a (mostly mild) Noonan syndrome. The variant was considered to be likely benign if real at all but extensively discussed with the parents in a formal counseling session. They decided against any further testing and the pregnancy is ongoing.

Drawing conclusions from the published evidence some caveats apply. Data currently available have been obtained using a variety of array platforms with low resolution BAC-based targeted arrays at one end of the spectrum and high resolution genotyping chips at the other. There is no formal agreement on a minimal level of resolution for microarrays used in prenatal diagnosis for the time being. The classification of VOUS and CNVs reported as pathogenic is not consistent in the different series and the assignment of a VOUS may be a matter of discretion (Figure 1), in particular if the parental genotype is unknown [38]. Considering the different clinical and diagnostic settings with labs serving highly specialized ultrasound units on the one hand and large commercial centers obtaining samples from a variety of sources on the other hand, patient ascertainment is likely to differ between the series as well. Regarding those heterogeneities and biases in data acquisition, we consider a meaningful comparison and stratification of reported CNVs and VOUS to be difficult and the drawing of firm conclusions premature at present. 

A positive correlation between array resolution and the detection rate of pathogenic CNVs as well as of VOUS is likely and not unexpected [39,40]. Srebniak *et al.* [41] have demonstrated this by retrospectively reassessing high resolution genome-wide array data of 456 fetuses at different resolution levels. They consider an implemented 0.5 Mb minimal detection threshold on a high resolution platform to be a favorable trade-off between the relevant criteria and propose this approach as a first-line prenatal cytogenetic test in cases without ultrasound abnormalities. This is an interesting model which, in contrast to the use of different platforms depending on the indication as applied by Shaffer *et al.* [42], implies the possibility to re-evaluate the data at a higher resolution if necessary e.g., in cases of ultrasound scan anomalies manifesting later in pregnancy. Approaches like these may finally pave the way for the replacement of conventional karyotyping by microarrays, but require careful pre-test information of the patients. The use of high resolution array designs in prenatal diagnosis was stated by most laboratories in a recent meeting report of the Genetic Services Quality Committee of the European Society of Human Genetics [9]. Targeted designs were considered to be disadvantageous because pathogenic imbalances may be missed and frequent updates of such platforms are required in order to include newly reported conditions. The authors also favor a common approach to post- as well as prenatal testing for most laboratories in order to gain a maximum of experience in data interpretation with a given platform.

Solid evidence accumulated in years of pre- and postnatal testing confirmed that microarrays reliably detect all copy number variation regardless of size within their technical limitations as discussed above. They will detect additional pathogenic CNVs as compared to conventional karyotyping in a proportion of cases depending on indication for testing and microarray resolution chosen. A preliminary reasonable estimate of the excess detection rate of microarrays in pregnancies with sonographic anomalies in a single anatomical system to be used for the counseling of affected parents might be around 5% rising up to 10% with multiple anomalies. Available data do not allow a meaningful and reliable stratification of detection rates according to specific sonographic findings at this time. The excess detection rate is significantly lower in pregnancies with an only moderate risk increase due to maternal age or a positive first trimester test result with 0.6% for known or potentially pathogenic CNVs in one major series [43], 1.7% (0.5% known pathogenic) in the US collaborative study [31] and 1.3% in a single center series of almost 400 patients (advanced maternal age exclusively) [37]. We are unaware of data for first trimester test results in the high positive range (e.g., >1:10). 

**Figure 2:**
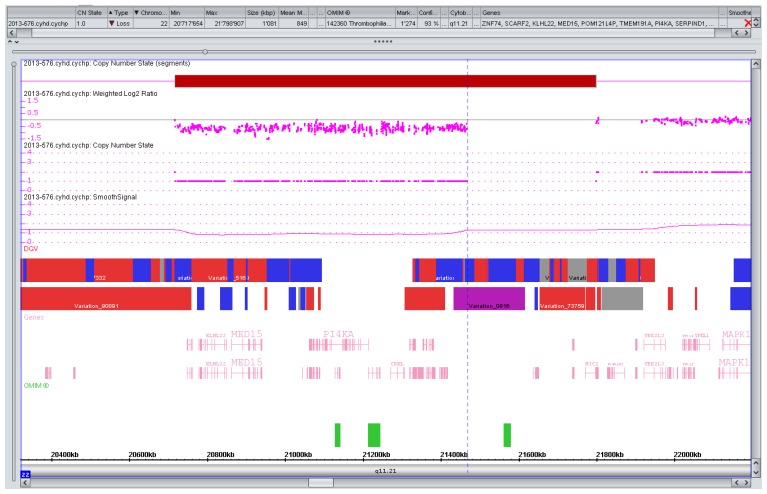
CNV with variable phenotype. Rare deletion (0.75 Mb) of the distal part of the 22q11 critical region for the DiGeorge/Velocardiofacial syndrome. Variable phenotypes have been reported [44]. The parents decided against further testing, the pregnancy is ongoing.

For reasons indicated earlier a sensible estimate of the frequency of VOUS is difficult. Published data suggest, however, that in low risk pregnancies VOUS might be more frequently encountered than true pathogenic CNVs at least with high resolution platforms [41]. The availability of parental blood samples is obviously a critical issue for the classification of copy number changes of doubtful significance and may reduce the VOUS rate dramatically [32] implying that a variant also present in a healthy parents is likely benign. This is usually reassuring to the parents and physicians but not always applicable. Increasing experience and a careful collection of data are expected to continuously ameliorate classification issues of genomic variation in the future. Well-defined CNVs with a variable prognosis such as the duplication of the Williams syndrome critical region [45] and others [6] (Figure 2) may also pose counseling challenges but are not specific to microarrays and well-known in classical chromosomal syndromes as well monogenic conditions. 

Unsolicited findings such as late onset inherited disorders or cancer predispositions are rare but of particular concern in the prenatal setting. Their frequency has been estimated as 1–2 per thousand [9] or 0.6% in a large postnatal cohort for CNVs affecting cancer genes [46].

### 3.2. Counseling Issues

High resolution karyotyping by microarrays is currently the most comprehensive approach to test for classical chromosomal disorders as well as submicroscopic copy number changes with a proven record of diagnostic accuracy. For some time, it has already been an essential extension to established diagnostic tools available in most specialized diagnostic labs and mainly offered in pregnancies at high risk for chromosomal conditions. Respecting patient autonomy [47] obliges to address microarray testing when discussing prenatal testing options in all pregnancies just as the new non-invasive aneuploidy tests. If microarray testing is considered pretest counseling must include information on the indication related expected detection rate, resolution specific VOUS rate as well as the possible need to test the parents. A clearly written agreement on the disclosure of unexpected or uncertain test results should be mandatory. 

### 3.3. The Local Approach to Prenatal Microarray Testing

We have introduced microarrays into prenatal cytogenetics having temporarily used a low resolution BAC based platform, but soon switched to a high resolution mixed oligo- and SNP array which we also use for postnatal testing. Our setting can be described as being a small academic lab working with a limited number of obstetricians highly specialized in maternal fetal medicine and serving mostly local patients. This overall setting allows for a close contact between the professionals involved as well as an immediate access to the patients. Comprehensive pre- and post-test counseling is provided. Our current policy is to:

- Highly recommend microarray testing for further characterization of abnormal results obtained by conventional chromosome analysis which are of unclear clinical significance such as *de novo* translocations, inversions, marker chromosomes and others.
- Recommend microarray testing in pregnancies at high risk for chromosomal abnormalities due to abnormal ultrasound findings or first trimester test results in the high positive range.
- Not encourage but accept the occasional parental request for a comprehensive exclusion of fetal chromosomal conditions even without a significant risk increase.
- Address microarray testing routinely with all patients seeking general advice on prenatal risk screening and testing options.

A general precondition for microarray testing is the parental consent to provide a blood sample if this is required to classify a CNV. Independent of insurance coverage issues the counseling approach is individualized, not strictly adhering to prefixed risk cut-offs which do serve, however, as a general orientation guide. One of these, relevant for insurance coverage, is the risk for “a fetal genetic condition” at a maternal age of 35 years. For practical implications, we consider risks smaller than this as low, risks beyond 2% as high and the range in between as intermediate. The access to microarray testing is not assigned but based on parental choice. 

The regular schedule includes 1 to 2 array runs per week allowing for a turn-around time of 7 to 10 days. Emergency testing within 3–4 days is available if required to provide a result before 24 completed weeks of gestation, the de facto limit for a termination of the pregnancy in this country. Microarray testing is usually carried out on uncultured amniotic fluid cells or chorionic villi if QF-PCR or direct chromosome preparation revealed a normal result. A backup culture is set up routinely. In cases with common aneuploidies a conventional chromosome analysis is initiated. We do not advocate further conventional testing if the microarray result was normal. Turn-around times for QF-PCR and direct chromosome preparation are 1–3 days and around 10 days for a conventional karyotype. In emergencies (high risk, late gestational age) microarrays are used as a first line test. Our current strategy is to report all known pathogenic deletions larger than 100 kb, duplication larger than 200 kb and findings of potential significance with regard to the individual reason for referral.

Patients undergoing microarray testing are aware of the possible occurrence of VOUS or other unsolicited findings. We do not ask for parental blood samples at the time of the invasive procedure. Should further testing be required this is discussed in a formal counseling session. Most parents agree to just have the CNV in question checked which has solved most VOUS issues we have had so far. CNVs with variable phenotypic consequences are always communicated and carefully discussed with the parents in analogy to e.g., an extra Y chromosome in conventional prenatal karyotyping. Pretest counseling includes also information on the unlikely event of detecting a predisposition for certain cancers or late onset disease. Written information, including an individual agreement on how to proceed with such information, has been in preparation for some time but will not be easily implemented into practice regarding the emotionally exceptional situation of many parents discussing a test after major fetal anomalies have been diagnosed. 

### 3.4. Should Microarrays Replace Conventional Karyotyping as a First-Tier Prenatal Diagnostic Test?

We believe that an entire replacement is premature and suggest adhering to the current approach shared by a majority of centers to not actively promote microarrays in low-risk pregnancies until more experience has been accumulated. We expect that a growing body of data on the clinical significance of copy number variation will help to prevent the provision of ambiguous information to our patients. The use of low-resolution targeted platforms to avoid the detection of VOUS will also miss pathogenic CNVs and may be a temporary solution for low-risk pregnancies but does not appear to be a promising long term option in particular for testing high risk pregnancies. Our own experience is in complete accordance with the recommendations by Vetro *et al.* [9] that a common platform for pre- and postnatal testing is advantageous in smaller labs. In our view this and other issues addressed earlier will enhance the trend towards high resolution platforms with genome wide coverage. Indication-specific or personal choice adaptation of the resolution level in such platforms [41] may be a viable option to increase the overall acceptability of molecular cytogenetics.

In principal, prenatal microarray testing does not alter the traditional dogma that invasive testing requires an increased risk for the conditions to exclude as practiced in some countries but not in others such as the US [48]. In health care systems with restricted access to invasive testing strictly adhering to risk cut-offs, the excess detection rate of microarray testing will have to be considered once reliable data are available. 

We do not advocate additional conventional karyotyping for economic reasons if the microarray result was normal being aware of missing an occasional balanced rearrangement some of which may pose a small risk for uniparental disomy. Abnormal microarray results must be followed-up by fetal and, if appropriate, parental karyotyping in order to exclude a parental balanced rearrangement implying a recurrence risk. Bui *et al.* [49] provide a more detailed account of the different views regarding the practical implementation of microarray testing. 

## 4. Conclusions

Microarrays are an established molecular tool for high resolution karyotyping in prenatal diagnosis. They are available as a variety of platforms with considerable differences in resolution and coverage of the genome. Their clinical utility is widely accepted in high risk pregnancies. The general replacement of conventional karyotyping as a first-tier clinical test also in pregnancies at low risk for chromosome anomalies is advocated by some but presently not supported by a majority of experts [7,8] mainly due to the detection of VOUS and other issues difficult to manage in a prenatal setting. While a change of policy may have been straightforward in postnatal cytogenetics, the prenatal setting proves to be incomparably more complex involving parental as well as fetal concerns, procedure related risks and method specific benefits and limitations. There is little doubt, however, that microarrays will eventually replace conventional karyotyping also in the prenatal setting in the near future, if invasive testing is required or requested. The novel non-invasive testing options, which appear to be gaining popularity, are expected to continuously restrict these requests to true high risk pregnancies.
